# Effect of Hole Arrangement on Failure Mechanism of Multiple-Hole Fiber Metal Laminate under On-Axis and Off-Axis Loading

**DOI:** 10.3390/ma14195771

**Published:** 2021-10-02

**Authors:** Jipeng Zhang, Yue Wang, Wen Yang, Yuan Zhao

**Affiliations:** 1 School of Transportation and Vehicle Engineering, Shandong University of Technology, Zibo 255000, China; zhangjp@sdut.edu.cn (J.Z.); yangwen004@sdut.edu.cn (W.Y.); 2 College of Engineering, Zhejiang Normal University, Jinhua 321004, China; zhaoyuan@zjnu.edu.cn

**Keywords:** fiber metal laminate (FML), multiple holes, failure mechanism, notched strength, off-axis load

## Abstract

Mechanical joints are commonly required in structures made of fiber metal laminate (FML), which pose a threat due to multi-site stress concentrations at rivet or bolt holes. Thus, for a reasonably designed FML joint, it is essential to characterize the failure mechanism of multiple-hole FML; however, little information about this has been found in open literature. In the present work, influences of hole arrangement and loading strategy (on-axis or off-axis) on the failure mechanism of multiple-hole FML were investigated, by performing finite element analyses and energy dissipation analyses with elastoplastic progressive damage models that took curing stress into account. Six types of specimens with holes arranged in parallel and staggered forms were designed, whose geometrical parameters were in strict accordance with those specified for composites joints. It indicated that the stress distribution, gross/net notched strength, critical fracture path, and damage evaluation process were only slightly influenced by the hole number and hole arrangement. On the other hand, they were strongly influenced by the loading strategy, due to the transition of failure domination. Results presented here can provide evidence for introducing design regulations of composite joints into the more hybrid FML, and for reasonably determining its multiple-hole strength merely based on the sing-hole specimen.

## 1. Introduction

Blunt notched behavior of composite has gain special attention in both the scientific and engineering communities, since complex mechanical responses and damage mechanisms are commonly encountered, and the higher notch sensitivity usually poses a threat to service security. To this end, extensive investigations have been carried out in this respect [1,2,3,4,5,6,7], in which specimens with central notches are generally adopted. In addition to those on the common fiber reinforced composites, a few investigations have also been performed on fiber reinforced metal laminate (FML), which consists of alternating layers of thin metal sheets and fiber reinforced composites [8].

In prior investigations, the effects of geometric parameters (notch shape and sizes of notch and specimen) [9,10,11,12,13,14], types and fractions of constituents [15,16,17], and interface adhesion [18] on mechanical response and damage behavior of FML with blunt notches were experimentally revealed. Moreover, finite element models [12,13,14,19,20,21,22] and analytical models, e.g., the point and average stress criteria [11,23] and effective crack growth model [24], were used to predict the notched strength, damage patterns, and damage evolution process. In addition to the common on-axis loading strategy, the influence of off-axis angle on notched behavior of FML was also investigated [12,13,14,16,21,22,25,26], where the evident off-axis dependence of notched strength, notch sensitivity, and failure mechanism were elaborated. Moreover, analytical methods for off-axis notched strength were also proposed based on the Norris theory criterion [25] and multi-axial criterion [26].

**Figure 1:**
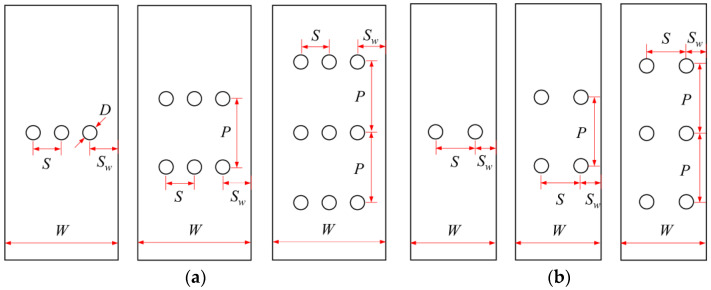
Multiple-hole FML specimens adopted in (**a**) [37] and (**b**) [38].

Blunt notches are generally presented as rivet or bolt holes in mechanical joints, and they usually appear in groups. Thus, it is inadequate to merely focus on the notched behavior of single-hole FML; more attention should be paid to those with multiple holes. In this regard, stress concentration near holes and their interactions in multiple-hole FML should be identified first, since these will give preliminary indications of the mechanical response and damage behavior. In past decades, stress concentration around holes in multiple-hole panels have been well discussed, such as those present in the fundamental books by Savin [27] and Pilkey [28], as well as those particularly present in fiber reinforced composite laminates [29,30,31,32,33]. In these investigations, the effects of the number and shape of holes, the relative distance between neighboring holes, the hole arrangement patterns, and the loading type on stress concentration were considered. However, it should be noted that the stress concentration behavior in multiple-hole FML has not been clearly identified. Moreover, although stress concentration in multiple-hole fiber reinforced composites has been previously investigated, research into their mechanical response and damage mechanism is still very limited to date [34,35,36,37,38]. Among them, only Chen et al. [37] and He et al. [38] paid attention to FML, where the specimens in Figure 1a and b were adopted, employing the geometrical parameters in Table 1.

Results from [37,38] show that FML with multiple holes do not differ much in gross notched strength, since net section areas remained constant and the large spacings between neighboring rows of holes could avoid interactions. The gross notched strengths of multiple-hole FMLs were also compared with those of the single-hole specimens that have the same widths, and higher values were obtained by the latter cases, due to the larger net section areas reserved. In consideration of the small hole spacings (*S*) in Figure 1, the gross notched strength reductions of multiple-hole FML could also be attributed to interactions between holes in the same row. In the net notched strength aspect, a reverse trend was obtained, i.e., the net notched strengths of multiple-hole FMLs were higher than those of the single-hole cases, which was a benefit from the larger fracture process zones in multiple-hole specimens [37]. Damage behavior of multiple-hole FML was also present, where fewer differences could be obtained between damage patterns and failure sequences of single- and multiple-hole FML, and the effect mechanisms of composite layer layup and off-axis angle also differed slightly between them. Beyond the detailed discussions in [37,38], further investigations on the failure mechanism of multiple-hole FML still need to be carried out, since the authors selected geometrical parameters related to hole arrangements shown in Table 1 but did not take into account the design specifications for composite joints. Moreover, the effect of staggered hole arrangements on the failure behavior of multiple-hole FML is still unknown, which is another typical joint configuration in engineering.

Within the scope of the present work, we focused on the failure mechanism of FML with multiple holes arranged in strict accordance with the specifications for fiber reinforced composite joints. We aimed at preliminarily identifying the availability of composite design regulations in FML, since the more hybrid FML has not been included in any composite design handbooks. Numerical investigations were carried out by employing finite element models, which were validated by experiments to be effective in simulating the notched behavior of FML. Initially, attention was paid to stress distributions along paths across the holes, as well as on the load bearing capacity characterized by gross and net notched strengths. These mechanical response results were further elaborated by discussing the final damage patterns and damage evolution processes, during which time the failure mechanisms were revealed. Six types of specimens referring to typical joint configurations were adopted, where both on-axis and off-axis cases were included, for consideration of the remarkable impact of off-axis load. The purpose was to elucidate the influence of hole arrangement on the failure mechanism of multiple-hole FML and its off-axis dependence, and to clarify whether the notched strength of multiple-hole FML could instead be given by that of the single-hole case.

## 2. Materials and Methods

### 2.1. Materials

**Figure 2:**
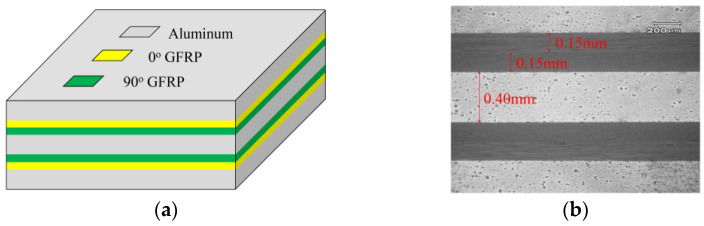
Configuration of the Glare: (**a**) Schematic view; (**b**) Cross-section view by SEM.

The FML adopted here was glass fiber reinforced aluminum laminate (Glare), which was consisted of 0.4mm 2024-T3 aluminum sheets and 0.15 mm S4C9 glass fiber reinforced SY24 epoxy composite (GFRP) prepregs. The mechanical properties of the aluminum sheet obtained from tensile tests are shown in Table 2, and those for the unidirectional GFRP laminates provided by the supplier are shown in Table 3, which were the same as those present in our previous investigation [21]. The relationships between the yield stress and plastic strain in Table 2 are based on the true stress–strain curve. As schematically shown in Figure 2a, the symmetric and orthogonal configuration was adopted, and a stacking sequence of [Al/0°/90°/Al/90°/0°/Al] was employed. An off-axis angle of 30° was selected to characterize the off-axis loading effect, for which the configuration of the specimen can be given as [Al/30°/−60°/Al/−60°/30°/Al]. The stacked Glare was cured in an autoclave for 2 hours under the conditions of 120 °C and 0.5 MPa; during this process, it was kept in vacuum bags. As shown by the cross-section view based on the scanning electron microscope (SEM) in Figure 2b, the as-cured Glare presented no impurities or debonding in the interfaces, which was a benefit from the phosphoric acid anodizing process conducted on the aluminum sheets prior to stacking.

### 2.2. Specimen Configurations

**Figure 3:**
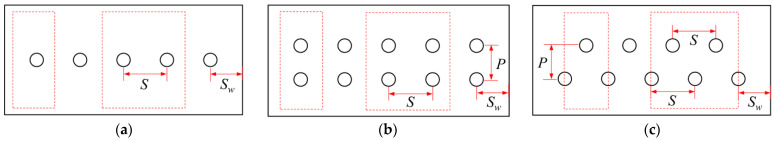
Typical joint configurations: (**a**) holes arranged in one row; (**b**) holes arranged in two rows with parallel form; (**c**) holes arranged in two rows with staggered form.

In engineering components, three types of mechanical joints (shown in Figure 3) are usually adopted, in which the joint holes are arranged in one or two rows with parallel and staggered forms. Generally, holes in the same row are uniformly distributed, i.e., the distance between neighboring holes in the same row (*S*) remains constant. On the basis of the extensive experiments on composite joints from the few past decades, the reasonable value of *S*, the pitch between neighboring rows (*P*), and the edge-to-hole distance (*S_w_*) have been determined in reference to the hole diameter (*D*). Proper values of *S*/*D*, *P*/*D*, and *S_w_*/*D* can be acquired from the handbook for joints of composites [39], as shown in Table 4. It should be noted that some other geometrical parameters are also required for the design of composite joints but are not present here, since they are out of the scope of the present investigation.

**Figure 4:**
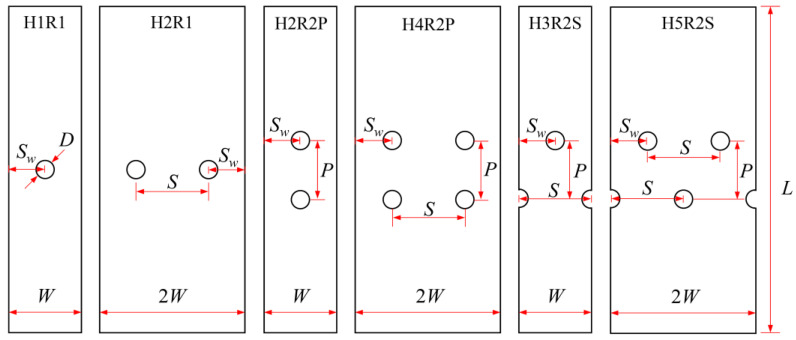
Schematics of notched Glare specimens with different hole arrangements.

Six types of notched Glare specimens concerning typical joint configurations were designed as shown in Figure 4, in which the hole patterns were centered in both the longitudinal and transverse directions. The specimens in Figure 4 were named based on the number of holes and hole arrangements (e.g., H4R2P denotes the specimen with four holes arranged in two rows with parallel form). The geometrical dimensions of these specimens are listed in Table 5, which are in strict accordance with those specified for composite joints in Table 4. As shown in Figure 4, these specimens can be seen as representative elements for the large joint configurations in Figure 3, which implies that results drawn from them will give exact indications for interactions between holes in Glare. Tensile tests at displacement control with a crosshead speed of 1 mm/min were conducted on the referential on-axis and off-axis H1R1 specimens, and post-failure analyses based on the microscope were also performed. Details about these have also been given in our previous investigation [21], which are presented here to provide evidence for the reliability of the finite element model in question. 

## 3. Finite Element Model

### 3.1. Materials Models

The two constituents in Glare, aluminum and GFRP, were modeled as isotropic elastic-plastic and orthotropic elastic materials according to their constitutive responses, respectively. The yielding of aluminum was predicted with the von Mises criterion, and its isotropic hardening behavior was assumed in the form of the relationship between yield stress and plastic strain [40], employing the elastic and plastic properties shown in Table 2. According to [19,37], which involved finite element modeling of Glare under tensile load, there is no need to define the fracture behavior of aluminum in Glare, while its damage evolution can be characterized by the equivalent plastic strain (PEEQ) instead. 

Damage evolution in GFRP usually plays an important or even a critical role in the failure process of Glare, with its lower ultimate strain compared to aluminum. In the present investigation, damage initiation in GFRP was predicted by Hashin’s criteria [41,42], which have been proved to be effective in simulating the damage behavior of GFRP in Glare [19,21,22]. Four failure modes, i.e., fiber tension (FT), fiber compression (FC), matrix tension (MT), and matrix compression (MC) as expressed in Equations (1)–(4), were defined in Hashin’s criteria. Once one of them was met, the damage evolution of GFRP was launched by degrading its stiffness and introducing damage variables into the elastic stiffness matrix [40].

(1) $$F_{f}^{t}={\left(\frac{\sigma_{11}^{eff}}{X^{T}}\right)}^{2}+{\left(\frac{\tau_{12}^{eff}}{S^{L}}\right)}^{2},\;\sigma_{11}^{eff}>0$$

(2) $$F_{f}^{c}={\left(\frac{\sigma_{11}^{eff}}{X^{C}}\right)}^{2},\;\sigma_{11}^{eff}<0$$

(3) $$F_{m}^{t}={\left(\frac{\sigma_{22}^{eff}}{Y^{T}}\right)}^{2}+{\left(\frac{\tau_{12}^{eff}}{S^{L}}\right)}^{2},\;\sigma_{22}^{eff}>0$$

(4) $$F_{m}^{c}={\left(\frac{\sigma_{22}^{eff}}{2S^{T}}\right)}^{2}+\left[{\left(\frac{Y^{C}}{2S^{T}}\right)}^{2}-1\right]\frac{\sigma_{22}^{eff}}{Y^{C}}+{\left(\frac{\tau_{12}^{eff}}{S^{L}}\right)}^{2},\;\sigma_{22}^{eff}<0$$

where $F_{f}^{t}$, $F_{f}^{c}$, $F_{m}^{t}$, and $F_{m}^{c}$ are damage factors; $\sigma_{11}^{eff}$, $\sigma_{22}^{eff}$, and $\tau_{12}^{eff}$ are effective stresses acting over the damage areas; and $X^{T}$, $X^{C}$, $Y^{T}$, $Y^{C}$, $S^{L}$, and $S^{T}$ are the strength parameters listed in Table 3.

In addition to the intralaminar damage, the interlaminar damage (delamination) serves as another key factor in the failure aspect of notched Glare, since it has been recognized that delamination in the vicinity of the notch can provide a benefit for stress redistribution [11,43]. In the present investigation, cohesive elements with bi-linear (linear elasticity prior to the onset of delamination and linear softening afterward) traction–separation constitutive responses were employed to simulate the delamination behavior. Given the possibility of mixed mode delamination, quadratic nominal stress and energy-based power-law criteria in Equations (5) and (6) [40] were adopted to predict the initiation and evolution of delamination:

(5) $${\left(\frac{\langle t_{n}\rangle }{t_{n}^{0}}\right)}^{2}+{\left(\frac{t_{s}}{t_{s}^{0}}\right)}^{2}+{\left(\frac{t_{t}}{t_{t}^{0}}\right)}^{2}=1$$

(6) $${\left(\frac{G_{n}}{G_{n}^{C}}\right)}^{2}+{\left(\frac{G_{s}}{G_{s}^{C}}\right)}^{2}+{\left(\frac{G_{t}}{G_{t}^{C}}\right)}^{2}=1\;$$

where *t_n_*, *t_s_*, and *t_t_* are tractions in normal and two shear directions in the current state, while those with superscript “0” correspond to their maximum values or interfacial strength. *G_n_*, *G_s_*, and *G_t_* are the work done by tractions in normal and two shear directions, while those with superscript “*C*” correspond to their critical values. The Macaulay bracket 〈 〉 (〈x〉=(x+|x|)/2) in Equation (5) indicates that delamination will not initiate under normal compressive load.

According to [44], the interfacial strength can be determined based on the GFRP properties, since no additional adhesive layer has been inserted. Thus, *t_n_*, *t_s_*, and *t_t_* in the present investigation are assumed to be equal to *Y^T^*, *S^L^*, and *S^T^* of GFRP, respectively, and the critical fracture energy release rates $G_{n}^{C}$, $G_{s}^{C}$, and $G_{t}^{C}$ were taken from [37]. These parameters of cohesive elements have been proven to be effective in characterizing the onset and evolution of delamination in the kind of Glare (composed with the same constituents) from our previous investigations [21,22]. 

### 3.2. Modeling Methods

**Figure 5:**
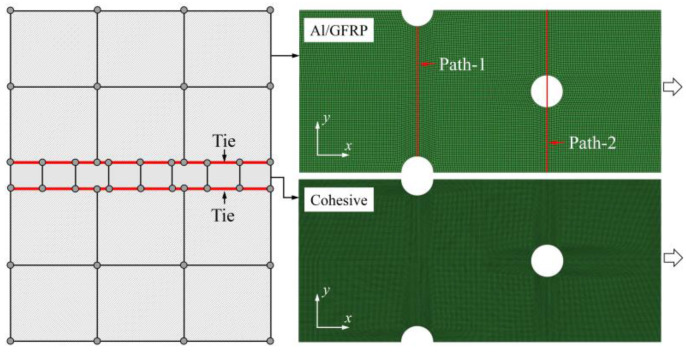
Mesh strategy and mesh refinements in vicinities of the holes (H3R2S specimen is an example; Path-1 and Path-2 were defined for stress distribution analyses).

Finite element analyses were performed on Abaqus by employing the explicit solver. As the configuration of the present Glare was symmetric in the thickness direction (Figure 2), only 1/2 models were built. The aluminum, GFRP, and interface parts were discretized with an eight-node linear brick solid element (C3D8R), eight-node quadrilateral continuum shell element (SC8R), and eight-node cohesive element (COH3D8), respectively. A mesh strategy (shown in Figure 5) was used to fulfill the mesh requirement for simulating delamination with cohesive elements, where the meshes of interface layers were more refined [45,46,47] and the tie constraints between them and coarse surrounding parts (aluminum and GFRP) were applied. For the entire model, mesh refinements were also applied in the vicinities of the holes, since high stress–strain gradients and damage initiations were more likely to take place there. The thermal expansion coefficients of aluminum and GFRP are rather different; thus, a predefined field prior to mechanical loading was applied to simulate the residual stress in the as-cured Glare. In this predefined field, the temperature decreased from 120 °C at the initial step to 25 °C at the following step, because residual stress for a thermoset composite is usually formed in the cooling process. Thereafter, a displacement load was applied at a reference point that coupled with one end of the specimen; meanwhile, all degrees of freedom at the opposite end were restricted. 

In addition to the results relating to stress, strength, and damage patterns, energy dissipation analyses were also carried out, due to their advantages regarding straightforwardly and effectively characterizing the mechanical behavior of FML [22]. The internal or total strain energy (*E*_int_) and damage dissipation energy (*E*_dam_) were required in both the laminate and constituent levels, where the latter case was implemented by creating individual element sets on aluminum, GFRP, and interface layers in the finite element model. 

## 4. Results and Discussions

### 4.1. Stress Distribution

**Figure 6:**
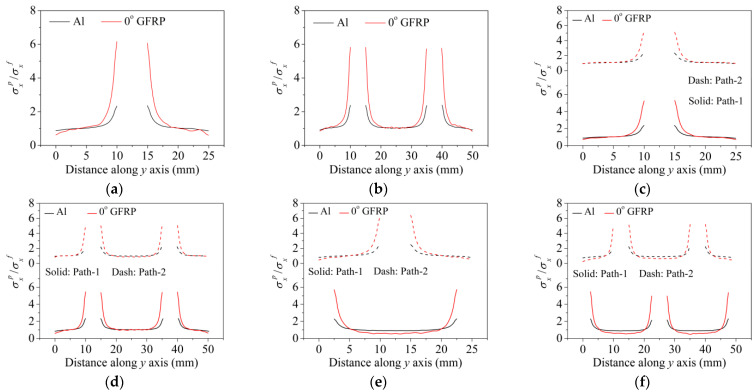
Axial stress distributions in on-axis Glare specimens: (**a**) H1R1; (**b**) H2R1; (**c**) H2R2P; (**d**) H4R2P; (**e**) H3R2S; (**f**) H5R2S.

**Figure 7:**
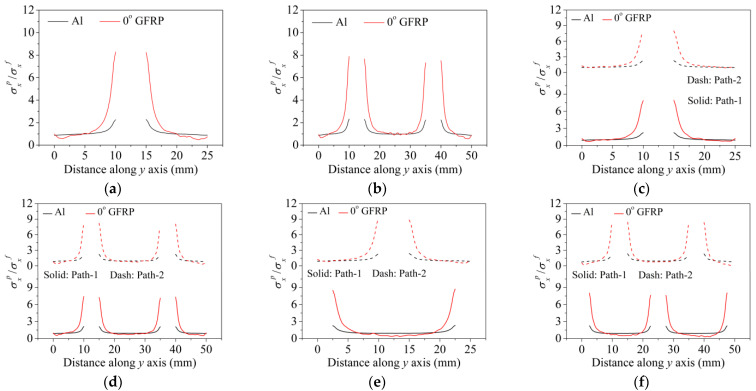
Axial stress distributions in off-axis Glare specimens: (**a**) H1R1; (**b**) H2R1; (**c**) H2R2P; (**d**) H4R2P; (**e**) H3R2S; (**f**) H5R2S.

In order to gain a preliminary understanding of interactions between holes in Glare, axial stress distributions in on-axis and off-axis specimens were revealed, as shown in Figure 6 and Figure 7, respectively. For the purposes of characterizing the stress concentration and reasonably comparing specimens with different hole arrangements, the stress in Figure 6 and Figure 7 is present in normalized form, i.e., axial stress along path across the holes ($\sigma_{x}^{p}$) is normalized by the axial far-field stress ($\sigma_{x}^{f}$). 

As shown in Figure 6 and Figure 7, the orthotropic nature of GFRP layers leads to more serious stress concentration than aluminum, which means GFRP layers will play an important role in the mechanical response of notched Glare, even though a lower GFRP volume fraction is adopted (33.33%). Irrespective of the stress concentration level along the hole edge, similar stress distribution patterns can be observed for a given specimen type in Figure 6 and Figure 7. Thus, the following discussions on stress distribution will not differ between on-axis and off-axis cases, while the off-axis effect will be illustrated in the following strength and damage analyses. 

From the stress distributions in the H2R1 and H4R2P specimens, it can be deduced that neighboring holes in the same row interact slightly for the present *S* adopted, since the stress concentration in between them is only a little higher than that away from them. Moreover, similar stress distributions are obtained in neighboring rows, when the holes are arranged in parallel form (H2R2P and H242P). By comparing the stress distribution pattern of one of the holes in a multiple-hole specimen to that of the single-hole case (H1R1), less of a difference can be observed. This phenomenon is consistent with the classic fundamental results for isotropic materials and composite laminates achieved previously. As shown by Savin for isotropic materials [27], the neighboring holes would not have an effect on each other if the distance between them was set to several times the hole diameter, nor would the number of holes have any influence. For holes aligned perpendicular to and along the loading direction, the proper distances were nearly 4 and 4.5 times the hole diameter, respectively. Also as shown by Xu et al. [31], who calculated the stress concentration in multiple-hole composite laminates by employing the complex potential method and adopting the Faber series expansion, the comformal mapping and the least squares boundary collocation techniques. It was concluded that when the distance between neighboring holes was more than 4.5 times the hole diameter in composite laminate, it would have influence less on the stress concentration, and then the laminate with multiple holes could be treated as that with a single hole. It should be noted that the stress concentration level for 0° GFRP in Figure 6 and Figure 7 is higher than that presented by Xu et al. [31], where the stress concentration was calculated for the whole laminate with a configuration of [0_4_/±45]_s_, and it was proven that the ±45° lamina benefitted decreasing the stress concentration. The above discussion suggests that the *S*/*D* and *P*/*D* parameters recommended for fiber reinforced composite may also be suitable for Glare. Also, they indicate that the notched behavior of Glare with multiple holes arranged in one row or two parallel rows may be represented by that of the single-hole case. This will be further validated in the following sections.

**Figure 8:**
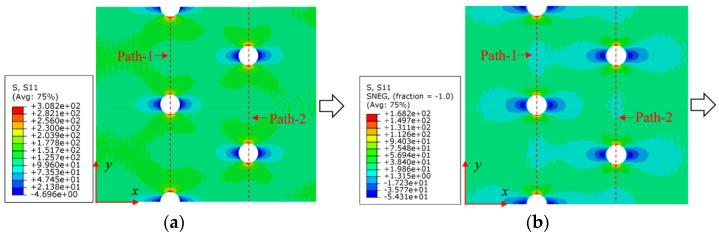
Axial stress distribution in constituents of on-axis H5R2S specimen: (**a**) aluminum; (**b**) 0° GFRP.

Interactions between holes in different rows can be observed for those arranged in staggered form (H3R2S and H5R2S), where the stress concentration in between holes in the the same row of GFRP layers is weakened, while that in aluminum is not. As shown in Figure 8, this is attributed to the lower stress regions below the holes having spread to the adjacent rows in GFRP layers, but those in aluminum layers are confined in small ranges. A similar phenomenon in GFRP laminate with multiple holes arranged in staggered form was presented in [35], in which the strain distribution was measured with digital image correlation (DIC). As shown by the DIC results in [35], the strain level in the vicinity of the hole was weakened at the position under the hole in the neighboring row. Looking at the stress distribution patterns, similar representations are obtained by the H3R2S and H5R2S specimens. In this regard, the H5R2S can indeed be treated as double replicates of H3R2S, and not just in the geometrical aspect. Moreover, from an overall perspective in Figure 6 and Figure 7, it can be deduced that the stress distribution around holes in Glare is slightly influenced by the hole arrangement, when distances between holes are determined as those specified for composite joints. This phenomenon is also similar to that presented Savin [27], who compared the stress concentration in an isotropic material with triangular and square hole arrangements, and less of a difference between them was observed. On the basis of the stress distribution in Figure 6 and Figure 7, it can be assumed that the arrangement of holes will not cause a serious impact on the notched behavior of Glare if geometrical parameters specified for composites joints are adopted. This will be further validated by the following notched strength and damage behavior analyses.

### 4.2. Notched Strength

**Figure 9:**
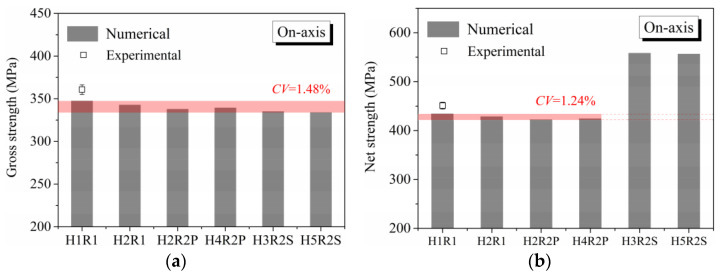
Effect of hole arrangement on notched strength of on-axis Glare: (**a**) Gross notched strength; (**b**) Net notched strength.

**Figure 10:**
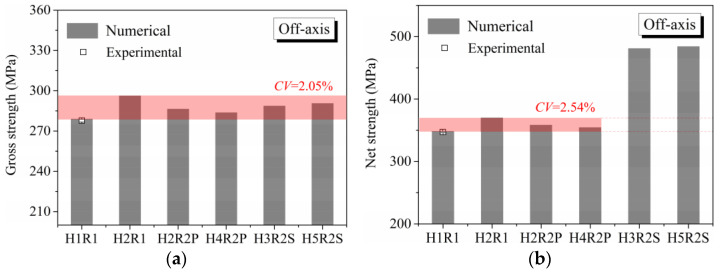
Effect of hole arrangement on notched strength of off-axis Glare: (**a**) Gross notched strength; (**b**) Net notched strength.

To clearly identify the effect of hole arrangement on multiple-hole Glare, notched strengths under on-axis and off-axis loading are present in Figure 9 and Figure 10, respectively, in which the gross and net notched strengths are calculated based on Equations (7) and (8).

(7) $$\sigma_{\mathrm{gross}}=\frac{P_{\max}}{Wt}$$

(8) $$\sigma_{\mathrm{net}}=\frac{P_{\max}}{W_{\mathrm{net}}t}=\frac{P_{\max}}{(W-nD)t}$$

where *σ*_gross_ and *σ*_net_ are gross and net notched strengths, *P*_max_ is the ultimate load achieved, *W* and *W*_net_ are gross and net section widths, *t* is the thickness, *D* is the hole diameter, and *n* is the number of holes that reduce the cross-section of the specimen. Therefore, *n* in specimens with holes arranged in parallel form (H2R2P and H4R2P) is half of the total number of holes.

As can be seen in Figure 9a and Figure 10a for gross notched strength, less of a difference between Glare with different hole arrangements can be observed, both for on-axis and off-axis loading conditions. Thus, it may be assumed that the mechanical responses of multiple-hole Glare can be represented by that of the single-hole case, regardless of the hole arrangement. Looking at the difference between gross notched strength of Glare with different hole arrangements for certain loading strategies, a more obvious variation is obtained by the off-axis case, as given by the comparison of their coefficients of variation (*CV*). This phenomenon is attributed to the different variations of damage patterns with hole arrangement in on-axis and off-axis loaded specimens, which will be specified in the following sections.

When the net notched strengths in Figure 9b and Figure 10b are focused, a similar appearance of *CV* in Figure 9a and Figure 10a can be obtained by specimens with parallel hole arrangements, i.e., the *CV* of net notched strength under off-axis load is higher than that under on-axis load. However, net notched strengths of specimens with staggered hole arrangements (H3R2S and H5R2S) do not behave as expected, where abnormal higher values are obtained. It may seem as though Glare with a staggered hole arrangement presents a higher load-bearing capacity than that with parallel form, but this is not the case in reality. As shown in Table 6, the ultimate loads of specimens with parallel and staggered hole arrangements are nearly the same when the same width and loading strategy are adopted. Then, the higher net notched strengths of H3R2S and H5R2S presented in Figure 9b and Figure 10b are just illusions, which in fact are caused by the calculation method of *W*_net_ in Equation (8). Thus, it can be concluded that the load-bearing capacity of multiple-hole Glare cannot be strongly influenced by the hole arrangement if geometrical parameters specified for composite joints are adopted. In other words, the designed notched strength of multiple-hole Glare can instead be given by that of the single-hole specimen, regardless of the hole arrangement. This phenomenon will be further validated by damage analyses in the following sections, where similar critical failure paths are obtained by specimens with parallel and staggered hole arrangements, i.e., the zig-zag fracture path along holes that indicates the stagger effect [35] does not appear. The results relating to notched strength in Figure 9 and Figure 10 are consistent with the results from Figure 6 and Figure 7, in which the stress distribution patterns in the vicinities of holes arranged in different forms are very similar. Comparisons between the experimental and numerical notched strengths of H1R1 specimens in Figure 9 and Figure 10 indicate that the proposed finite element model is able to predict the notched behavior of Glare well; details about this were also presented in our previous publications [21,22], in which the same modeling approach was adopted.

For revealing the off-axis dependence of the notched strength of multiple-hole Glare, a parameter called the coefficient of off-axis sensitivity (*C*_OAS_) was defined in Equation (9). It should be noted that *C*_OAS_ does not vary depending on whether the gross or net notched strengths are adopted, because the value of *σ*_on-axis_/*σ*_off-axis_ is equal to that of *P*_on-axis_/*P*_off-axis_ for a given specimen configuration.

(9) $$C_{\mathrm{OAS}}=1-\frac{\sigma_{\mathrm{off}-\mathrm{axis}}}{\sigma_{\mathrm{on}-\mathrm{axis}}}$$

where *σ*_on-axis_ and *σ*_off-axis_ are gross or net notched strengths under on-axis and off-axis load, respectively.

**Figure 11:**
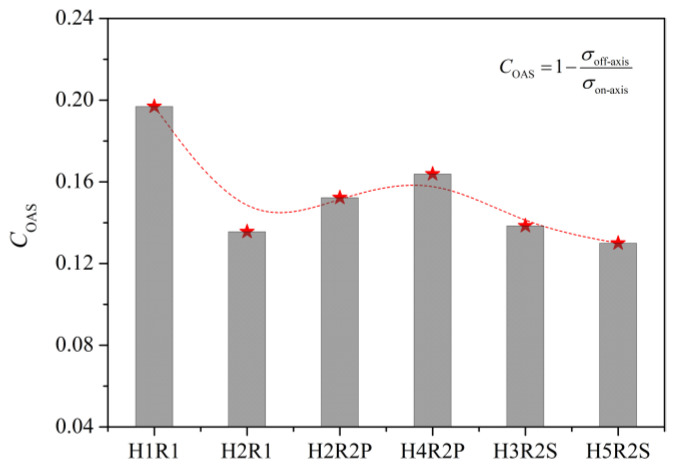
Effect of hole arrangement on off-axis dependence of notched strength of Glare.

**Figure 12:**
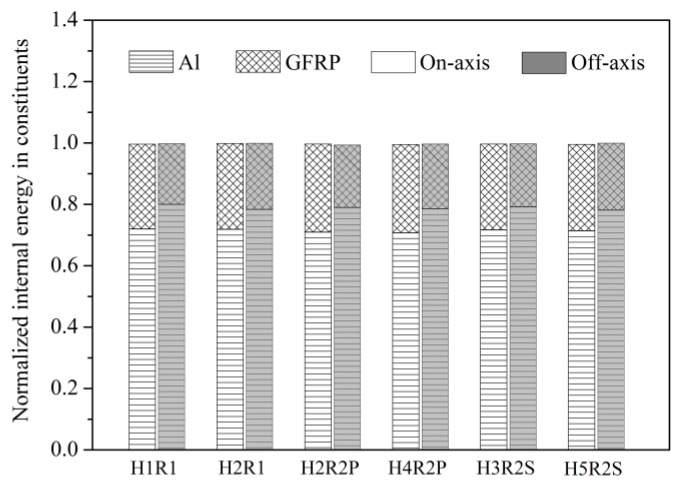
Normalized internal energy in constituents of Glare with different hole arrangements.

**Figure 13:**
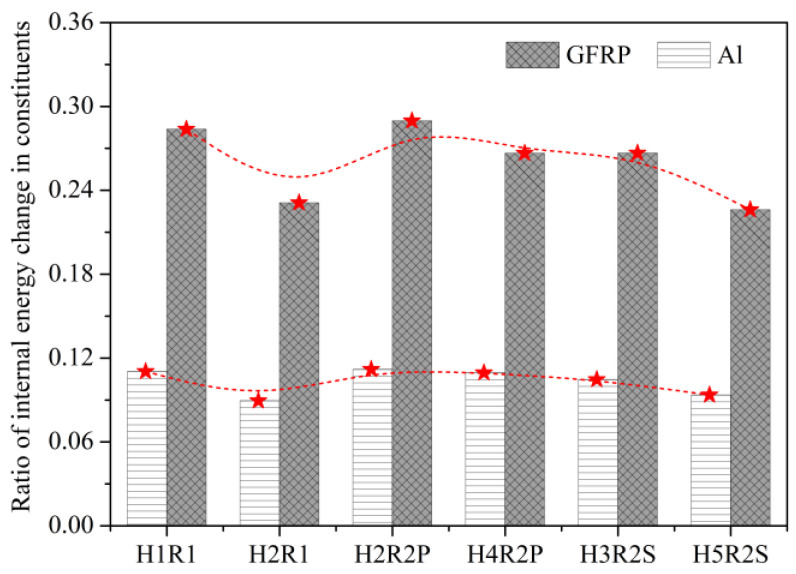
Ratios of internal energy change in constituents of Glare induced by off-axis load.

As shown in Figure 11, the *C*_OAS_ value of notched Glare can be influenced by the hole arrangement, but the variations are confined to a small range of 0.13–0.20. This is attributed to the large volume fraction of aluminum (66.67%) in the present Glare, which is less sensitive to the off-axis load. It will be very difficult or even impossible to clarify the off-axis dependence of the notched strength of Glare, or its complicated relationship with the hole arrangement, by merely focusing on stress, strain, or damage patterns. To this end, the energy dissipation approach is adopted for interpretation in Figure 12 and Figure 13, following the belief that any mechanical responses should obey the energy principles.

As shown in Figure 12, imposing the off-axis effect on Glare can lead to clear changes of internal energies in aluminum and GFRP layers. In view of this, the variation of *C*_OAS_ in Figure 11 may be elucidated by the ratios of internal energy change in Glare. As expected, in Figure 13, the variation trends of ratios of internal energy change in GFRP and aluminum layers are similar to that of *C*_OAS_, which suggests that the off-axis dependence of the notched strength of Glare is closely associated with the work done by constituents. It also confirms the perspective that the mechanical behavior of the hybrid FML can be straightforwardly and effectively characterized from the energy dissipation point of view.

### 4.3. Damage Behavior

**Figure 14:**
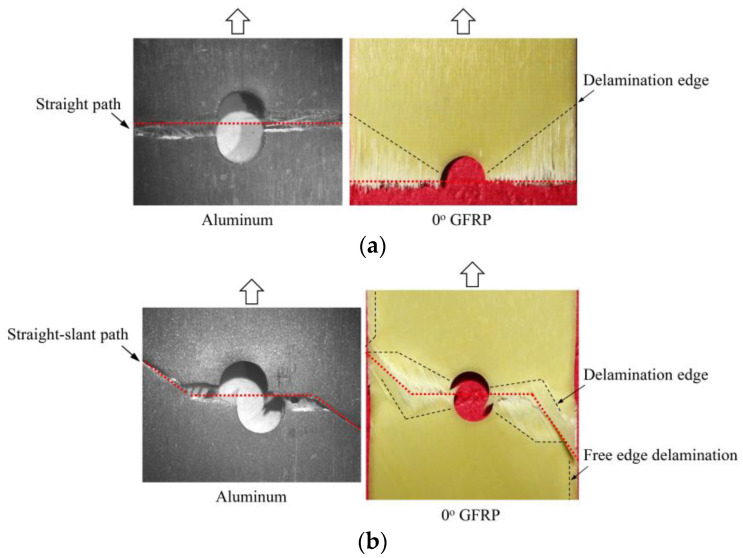
Experimental damage patterns of H1R1 Glare laminate [21]: (**a**) On-axis; (**b**) Off-axis.

To further reveal the interactions between holes in Glare, and to validate the above conclusions drawn from stress distribution and notched strength analyses, damage patterns of on-axis and off-axis Glare with different hole arrangements are presented in Table 7 and Table 8, respectively, in which the schematic critical fracture paths that play dominant roles in the failure aspect are also given for clarity. The reliability of the numerical damage patterns can be validated by our previous experimental results of on-axis and off-axis H1R1 specimens [21] in Figure 14, where better agreements with those shown in Table 7 and Table 8 are achieved. It should be noted that damage patterns of the 90° composite layers are not presented here, since they are slightly loaded and less critical in this cross-ply Glare.

As can be seen in Table 7, critical fracture paths in on-axis Glare are all present in transverse form, regardless of the hole arrangement. This transverse fracture behavior is characterized by the failure throughout of aluminum and 0° GFRP layers in the same row in the width direction; they were detected as PEEQ and fiber tension breakage, respectively. Along these critical fracture paths, extensive delaminations were observed, and they also spread throughout the width. In the non-critical row, obvious failure in aluminum, 0° GFRP, and interfaces were also observed. These multiple-site damages in addition to the critical fracture path can weaken the load-bearing capacity as well, which then leads to the slightly lower notched strength of multiple-hole Glare in Figure 9. It should be noted that damages in different rows do not interact with each other where the critical fracture path across one hole (H2R2P, H3R2S) is similar to that of the single-hole specimen(H1R1), while those across two holes (H2R1, H4R2P, H5R2S) can be treated as two duplicates. This phenomenon is consistent with the results from the stress distribution in Figure 6, and it is the very reason that the notched strengths of on-axis multiple-hole Glare are very close to that of the single-hole case.

When the off-axis effect is imposed, drastic changes of damage patterns of notched Glare are obtained. As seen from Table 8, critical fracture paths of off-axis notched Glare are characterized by transverse straight fractures in vicinities of holes and slant fractures towards the free edges, where, uniquely, the failure of GFRP layers are dominated by both the fiber tension breakage and matrix shear-off. Similarly to the on-axis case, damages also appear in vicinities of non-critical holes, but they are so slight that they cannot pose a threat to the load-bearing capacity. The multiple-hole Glare presented a slightly higher notched strength than the single-hole case under off-axis load, taking the benefits of the well-developed non-critical damages that redistribute the stress. As for the prominent H2R1 and H5R2S specimens, broad delamination was detected in between holes along the fracture path. Since remarkable propagation of delamination can accommodate the stress concentration [11,43], the matrix shear-off, which acts as the final defense of the GFRP layer, is postponed, and then extensive fiber breakages are obtained by H2R1 and H5R2S. The longer fiber breakage length in the off-axis loaded GFRP layer can indicate its higher load-bearing capacity [21], which consequently results in the slightly higher notched strength of multiple-hole Glare under off-axis load. The most special damage pattern under off-axis load was obtained by the H4R2P specimen, where the fracture path passed through two holes in different rows. It may seem as though a zig-zag fracture pattern was obtained, but the fact is that the fiber-aligned matrix shear damages had spread into each other. 

Compared with the on-axis case, damage patterns of the off-axis Glare with different hole arrangements differ more substantially, which then results in the relative higher *CV* of notched strength in Figure 10. Nevertheless, it still remains at a lower level, which is attributed to the fact that failure of notched Glare under off-axis load is dominated by the aluminum [21], due to its excellent shear resistance in comparison with GFRP. It is also the reason that the critical fracture paths in Table 8 are similar. The damage patterns in Table 7 and Table 8 provide further evidence for the above statements, i.e., notched strength of multiple-hole Glare can instead be specified by that of the single-hole specimen, and it is also not heavily dependent on the hole arrangement, if geometrical parameters recommended for composite joints are adopted.

**Figure 15:**
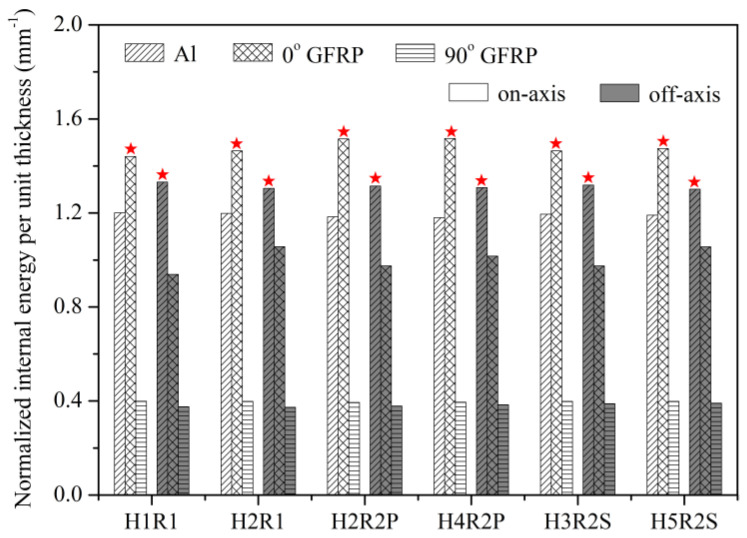
Normalized internal energy per unit thickness in constituents of Glare with different hole arrangements.

The different failure mechanisms of multiple-hole Glare under on-axis and off-axis loading can be preliminarily identified by energy dissipations, which is very intuitive. As in Figure 15, normalized internal energy per unit thickness (*E*_int_/*t*) in aluminum and GFRP layers are compared, demonstrating that the highest values of *E*_int_/*t* were achieved by 0° GFRP layers under on-axis load, while for off-axis cases, they were achieved by aluminum layers instead. The normalized internal energy in aluminum and GFRP layers provided reasonable comparisons of the work done by them in the loading process, and further elimination of the thickness effect can indicate their contributions to load-bearing. Thus, the results relating to *E*_int_/*t* in Figure 15 imply that failure of on-axis and off-axis notched Glare is dominated by 0° GFRP and aluminum, respectively, and as expected, this transition of failure mechanism cannot be influenced by the hole arrangement.

**Figure 16:**
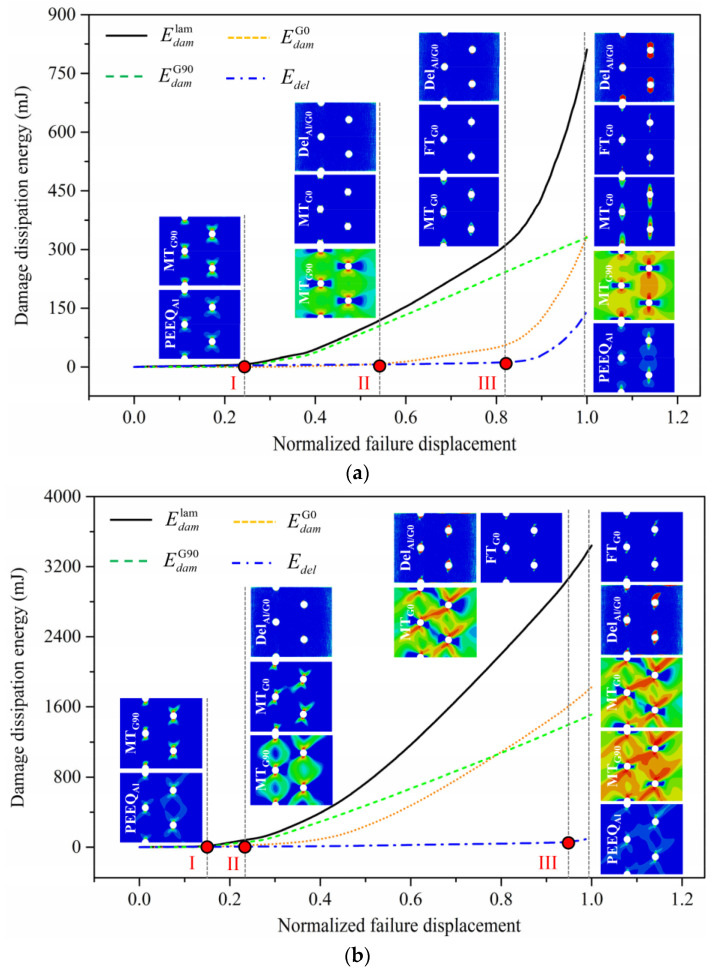
Damage evolution characterization of H5R2S specimen, employing the energy dissipation approach: (**a**) On-axis; (**b**) Off-axis.

Details about the failure mechanism should be revealed based on the progressive damage analysis. In this regard, damage evolution processes of multiple-hole Glare are presented in Figure 16, in which the H5R2S specimen is given as an example. The damage evolution is characterized by damage dissipation energy in the loading process, including those for 0° GFRP ($E_{dam}^{G0}$), 90° GFRP ($E_{dam}^{G90}$), and interfaces ($E_{del}$), as well as that for the hole laminate ($E_{dam}^{\mathrm{lam}}$) equal to the total of the former three. In addition to them, damage patterns at some feature points marked with capital roman numerals are also presented.

As seen in Figure 16a for the on-axis case, damage in GFRP took place firstly in 90° layers in the form of matrix damage at the early stage of the loading process (point I), and at this point, plastic deformation in aluminum had initiated as well. The matrix damage in 90° GFRP developed rapidly in the following loading process, which is expressed by the higher slope of the $E_{dam}^{G90}$ curve. When nearing the middle stage (point II), matrix damage in 0° GFRP was detected; meanwhile, slight delamination was also observed from the damage cloud, though it is not so obvious in the $E_{del}$ curve. With the loading process going forward, matrix damage in 0° GFRP and interfacial delamination intensified, but they were not so serious until fiber tension damage in 0° GFRP was acquired at point III. In the later short loading process after fiber breakage initiated, rapid growths of the $E_{dam}^{G0}$, $E_{del}$, and $E_{dam}^{\mathrm{lam}}$ curves were achieved, and towards the failure point, serious fiber breakage in 0° GFRP and plastic deformation in aluminum were detected. This suggests that failure of multiple-hole Glare under on-axis load is dominated by fiber breakage in 0° GFRP. 

For the off-axis case in Figure 16b, matrix damage in 90° GFRP and plastic deformation in aluminum were initially observed, but soon after that, matrix damage in 0° GFRP and delamination were observed as well. In the latter loading period, these subcritical damages developed more seriously compared to those in the on-axis case, which is a benefit for the stress accommodation; then, fiber breakage was observed until the loading process approached the failure point. It is worth noting that remarkable shifts of the energy curves after fiber breakage initiation did not appear here as in the on-axis case; also, the damage degrees at the failure point were not so serious. This phenomenon reveals that failure of multiple-hole Glare is not merely dominated by fiber breakage under the tension–shear stress state induced by the off-axis load, but also by the aluminum, due to its relatively higher shear resistance. The damage evolution process and failure mechanism of multiple-hole specimens are similar to those of the single-hole specimens observed in our previous investigations [21,22]. This further confirms that the notched strength of multiple-hole Glare for a certain loading strategy can instead be given by that of the single-hole specimen, regardless of the hole arrangement.

## 5. Conclusions

Failure mechanisms of multiple-hole Glare laminates under on-axis and off-axis tensile loading were investigated. Parallel and staggered hole arrangements were designed in reference to configurations of mechanical joints commonly used in engineering structures, and the critical geometrical parameters adopted were those specified in the handbook for composite joints.

Here, it was shown that multi-site stress concentrations in the vicinities of the holes interacted slightly under both the on-axis and off-axis loading, and stress distribution patterns around holes in multiple-hole specimens were even similar to that of the sing-hole case. This gave preliminary indications that the design values of notched strength of multiple-hole Glare may be represented by that of the single-hole specimen. As expected, this was confirmed by analyzing notched strength, where gross notched strength differed slightly between different hole arrangements, including in comparison with the single-hole specimen. Similar results were also achieved when net notched strength was examined. Aside from the illusions appearing in specimens with staggered hole arrangements (caused by the calculation method of net section width), the load-bearing capacity did not greatly vary. Evidence for this was provided by further investigating the damage behavior, where similar critical fracture paths were obtained by specimens with different hole arrangements for a certain loading strategy. Based on the available energy outputs in the finite element model, damage evaluation mechanisms of multiple-hole Glare under on-axis and off-axis loading were revealed, where the transition of the failure domination was achieved. This gave rise to the off-axis dependence of notched strength, but it also slightly varied with the hole arrangement. 

The present results suggest that the FML joint can be designed to be similar to the fiber reinforced composite joint. They also indicate that design values of notched strength of multiple-hole FML can instead be determined by that of the single-hole specimen if the holes are arranged under regulations specified for the composite joint. This will provide benefits relating to saving cost and time in design and compliance verification stages.

## Figures and Tables

**Table 1:** Geometrical parameters of specimens in [37,38].

Parameter	*W* (mm)	*D* (mm)	*S* (mm)	*P* (mm)	*S_w_* (mm)	*S*/*D*	*P*/*D*	*S_w_*/*D*
[37]	40	4	15	50	5	3.75	12.5	1.25
[38]	25	4	12	50	6.5	3	12.5	1.625

**Table 2:** Mechanical properties of 2024-T3 aluminum sheet [21].

Young’s Modulus (GPa)	Poisson’s Ratio	Yield Stress (MPa)	Plastic Strain	Yield Stress (MPa)	Plastic Strain
70.42	0.33	321.88	0	450.01	0.04295
		340.09	0.00011	470.05	0.05551
		360.05	0.00308	490.01	0.07028
		380.06	0.01002	510.00	0.08771
		400.05	0.01813	530.02	0.10928
		420.04	0.02721	542.85	0.12518
		430.00	0.03207		

**Table 3:** Mechanical properties of unidirectional GFRP laminate [21].

Parameter	Value	Unit
Longitudinal stiffness $E_{1}$	54.6	GPa
Transverse stiffness $E_{2}=E_{3}$	10.5	GPa
Shear stiffness $G_{12}=G_{13}$	5.5	GPa
Shear stiffness $G_{23}$	3.9	GPa
Poisson’s ratio $\nu_{12}$	0.33	-
Longitudinal tensile strength $X^{T}$	1850	MPa
Longitudinal compressive strength $X^{C}$	1037	MPa
Transverse tensile strength $Y^{T}$	62.2	MPa
Transverse compressive strength $Y^{C}$	144	MPa
Longitudinal shear strength $S^{L}$	129	MPa
Transverse shear strength $S^{T}$	76.1	MPa

**Table 4:** Geometrical parameters specified for composite joints [39].

Parameter	*S*/*D*	*P*/*D*	*S_w_*/*D*
Value	≥5	≥4	≥2.5

**Table 5:** Dimensions of notched Glare specimens in present investigation.

Parameter	*L* (mm)	*W* (mm)	*D* (mm)	*S* (mm)	*P* (mm)	*S_w_* (mm)	*S*/*D*	*P*/*D*	*S_w_*/*D*
Value	150	25	5	25	20	12.5	5	4	2.5

**Table 6:** Comparisons between ultimate loads of multiple-hole Glare with holes arranged in parallel and staggered forms (loads were achieved by 1/2 symmetric models in the thickness direction).

Specimen	*W*_tot_ (mm)	*W*_net_ (mm)	*P*_on-axis_ (kN)	*P*_off-axis_ (kN)
H2R2P	25	20	7.60	6.46
H3R2S		15	7.54	6.50
H4R2P	50	40	15.27	12.77
H5R2S		30	15.03	13.08

**Table 7:** Damage patterns of on-axis Glare with different hole arrangements.

Notched Specimens	Types of Damage				Schematics of Critical Fracture Path
	PEEQ_Al_	FT_G0_	MT_G0_	Del_Al/G0_	
H1R1 [21]	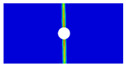	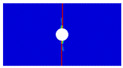	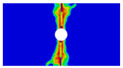	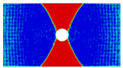	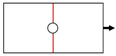
H2R1	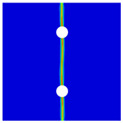	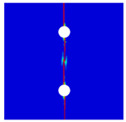	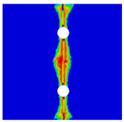	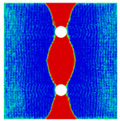	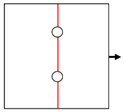
H2R2P	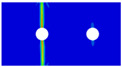	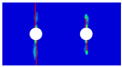	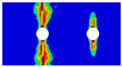	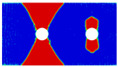	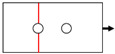
H4R2P	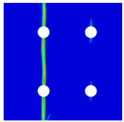	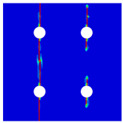	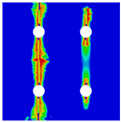	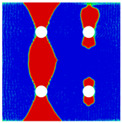	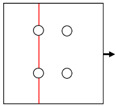
H3R2S	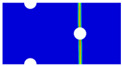	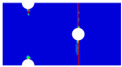	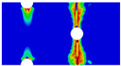	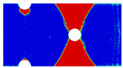	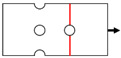
H5R2S	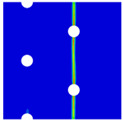	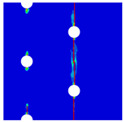	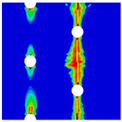	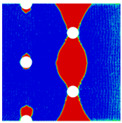	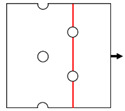

**Table 8:** Damage patterns of off-axis Glare with different hole arrangements.

Notched Specimens	Types of Damage				Schematics of Critical Fracture Path
	PEEQ_Al_	FT_G0_	MT_G0_	Del_Al/G0_	
H1R1 [21]	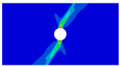	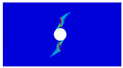	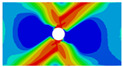	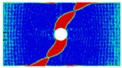	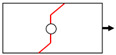
H2R1	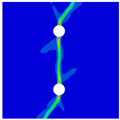	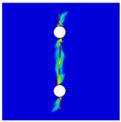	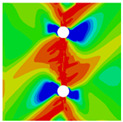	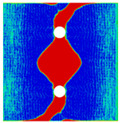	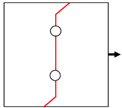
H2R2P	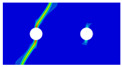	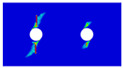	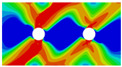	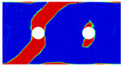	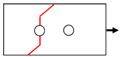
H4R2P	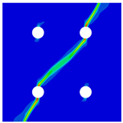	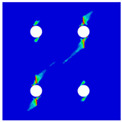	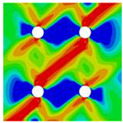	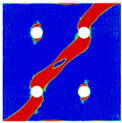	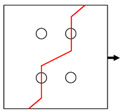
H3R2S	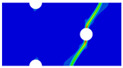	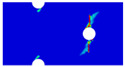	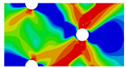	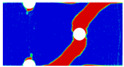	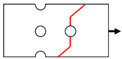
H5R2S	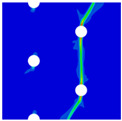	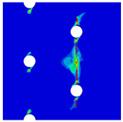	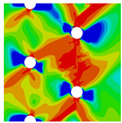	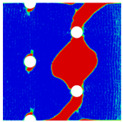	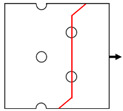

## Data Availability

The data cannot be shared at this time as they also forms part of an ongoing study.
